# Early detection of contractile dysfunction in GRMD dogs by post-processing of standard cine FLASH-MRI

**DOI:** 10.1186/1532-429X-13-S1-P337

**Published:** 2011-02-02

**Authors:** Raymond Gilles, Jean-Laurent Thibaud, Marcel Toussaint, Stéphane Blot, Pierre G Carlier

**Affiliations:** 1 CHWAPI, Tournai, Belgium; 2 Ecole Nationale Vétérinaire d'Alfort, Alfort, France; 3 CH Sud Francilien, Corbeil-Essonnes, France; 4 Institut Myologie / Pitié-Salpétrière and CEA, Paris, France

## Introduction

Duchenne muscular dystrophy (DMD) due to dystrophin deficiency leads to death by heart failure in nearly 30% of cases. An early detection of myocardial abnormalities in these patients would help in the optimization of their management. The GRMD is a canine model of DMD, that develops a severe cardiomyopathy.

## Purpose

Post-processing of standard cine-MR images of young GRMD dogs, before any sign of cardiac global dysfunction is patent. The aim of the study was to detect minor changes in synchronism or contraction patterns of the left ventricle of golden retriever muscular dystrophy (GRMD) dog as compared to control dogs.

## Methods

6-month old GRMD (n=5) and control dogs (n=4) were imaged using a Siemens Magnetom Trio TIM with a standard cine-FLASH sequence in short axis. Acquisition parameters were as follows: TE:3.06ms, TR:19.74ms, flip angle:15°, slice thickness:6mm, in plane resolution:1.8x1.8mm^2^, NEX:3, GRAPPA:2. Each dog heart was covered by 8 slices. We used the Segment® software to define the endo- and epicardial contours and analyze radii and wall thicknesses in each slice with a 6 segment model. The radii and wall thickness curves were smoothed using a Savitzky-Golay filter. For each slice, a dyssynchrony index was calculated, as the SD of the normalized radii across the six segments throughout the cardiac cycle. Max dRadius/dt and max dWT/dt were also extracted for each segment of each slice.

## Results

**Figure 1:**
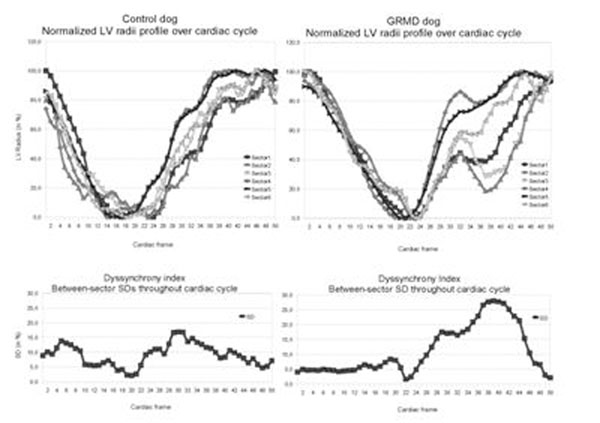
Upper panel: Examples of basal slice radius curves in a control dog (left) and a GRMD (right) throughout cardiac cycle. Lower panel: corresponding time-courses of dyssynchrony index.

**HR,** LVEF, peak ejection rate and SV were not statistically different between GRMDs and controls. EDV was somewhat smaller in GRMDs (46.0±8.4 vs 34.7±5.2 ml ; p=0.04). The GRMDs showed an higher mean dyssynchrony index of the 4 most basal slices lower than the controls (11.5±1.9 vs 9.7±2.3%; p=0.015). Max dRadius/dt during contraction and during relaxation was higher in GRMDs than in controls (58±21 vs 37±12 mm/s; p<0.001) and (63±25 vs 46±16 mm/s; p<0.001). The same appeared true for max dWT/dt in contraction and relaxation (33±16 vs 116±109 mm/s; p<0.001 and 43±20 vs 119±132 mm/s; p<0.001 for controls vs GRMDs). Figure 1.

## Conclusions

Pre-clinical contractile abnormalities can be detected by post-hoc analysis of standard cine MR images in GRMD dogs. This preliminary results should encourage further work on early detection of myocardial minimal dysfunction in GRMD dogs but also in DMD and Becker muscular dystrophy patients.

